# Extraction of Fenugreek (*Trigonella foenum-graceum* L.) Seed Oil Using Subcritical Butane: Characterization and Process Optimization

**DOI:** 10.3390/molecules22020228

**Published:** 2017-02-02

**Authors:** Ling-Biao Gu, Xiao-Ning Liu, Hua-Min Liu, Hui-Li Pang, Guang-Yong Qin

**Affiliations:** 1 School of Physics and Engineering, Zhengzhou University, 100 Science Avenue, Zhengzhou 450001, Henan Province, China; gulingbiao@foxmail.com (L.-B.G.); liuxiaoning@gs.zzu.edu.cn (X.-N.L.); qinguangyong@zzu.edu.cn (G.-Y.Q.); 2 College of Food Science and Technology, Henan University of Technology, 100 Lianhua Road, Zhengzhou 450001, Henan Province, China

**Keywords:** fenugreek seed oil, subcritical butane extraction, response surface methodology, fatty acid

## Abstract

In this study, the subcritical butane extraction process of fenugreek seed oil was optimized using response surface methodology with a Box-Behnken design. The optimum conditions for extracted oil from fenugreek seed was as follows: extraction temperature of 43.24 °C, extraction time of 32.80 min, and particle size of 0.26 mm. No significant differences were found between the experimental and predicted values. The physical and chemical properties of the oil showed that the oil could be used as edible oil. Fatty acid composition of oils obtained by subcritical butane under the optimum conditions and by accelerated solvent extraction showed negligible difference. The oils were rich in linoleic acid (42.71%–42.80%), linolenic acid (26.03%–26.15%), and oleic acid (14.24%–14.40%). The results revealed that the proposed method was feasible, and this essay shows the way to exploit fenugreek seeds by subcritical butane extraction under the scope of edible oils.

## 1. Introduction

Fenugreek (*Trigonella foenum-graceum* L.) is a self-pollinating annual herbaceous aromatic leguminous crop, also known as bird’s foot, Greek hayseed, and methi [1]. It is now widely cultivated in northern Africa, Europe, west and south Asia, north America, Argentina, and Australia [2]. Fenugreek is considered the oldest known medicinal plant in human history [3]. It was used for the treatment of diabetes and also has been utilized as a galactogogue.

Fenugreek seed is a good source of essential amino acids, especially leucine, lysine, and total aromatic amino acids. Recently, researchers have found that the seed contains 20%–25% protein, 6%–8% oil, 45%–50% dietary fiber [4], and 2%–5% steroidal saponin [5]. The seed is well characterized with a distinctive pungent scent that impacts flavor, color, and aroma of foods, making it highly desirable in culinary applications as a food spice in countries where it is grown [6]. Advances in nutraceuticals and demand for functional foods have stimulated interest in fenugreek as a functional food. An increase in demand for food implies the need to increase the production of alternative sources of edible oils. Therefore, this study was focused on the fenugreek seed oil, which has many health benefits. Fenugreek seed oil (mainly of unsaturated acids, namely linoleic, linolenic, and oleic acid) [7], is used in flavoring many canned foods and syrups and as an ingredient in soma perfumes [6]. Schuette et al. investigated the fenugreek seed oil obtained by Soxhlet extraction using petroleum ether as a solvent, and a yield of 6.7% was obtained [8]. Ren et al. studied the optimal conditions for the extraction of oil from fenugreek seed by supercritical CO_2_ fluids. The highest extraction yield under the optimum condition was found to be 8.95% [7]. Arivalagan et al. determined the seed oil content in fenugreeks of different genotypes; the oil content ranged from 3.25% to 6.88% among 46 accessions of fenugreek genotypes [9]. Savitha et al. studied the effect of grinding on the yield of fenugreek seed oil using Soxhlet extraction; the results implied that fractions less than 200 μm produced the highest oil yield of about 8% [5].

Conventional oil extraction technologies mainly include chemical extraction and mechanical pressing. Chemical extraction processes are dangerous to handle and unacceptable as they employ solvents such as hexane, which are very noxious to environment and human health. The mechanical pressing process consumes a large amount of energy, and the extraction yield is low [10]. Supercritical CO_2_ extraction appears to be an innovative method, which has advantages such as being nontoxic, leaving zero residual organic solvent, and allowing for more selective extractions [11]. However, high pressure and long extraction time are required. Accelerated solvent extraction (ASE) is a novel automated oil extraction technique which employs low-boiling solvents or solvent mixtures at a high-temperature (up to 200 °C) and pressure (up to 3000 psi) to reduce extraction time and solvent usage. It has been proven to be an effective technique for the extraction of lipid from seeds [12]. Thus, it can be used for comparison as a standard to determine the efficiency of other methods on the oil yield [13]. 

One alternative method is subcritical butane extraction (SBE), which has several advantages besides the merits of all conventional technologies. The extraction process is safe and efficient, has good selectivity and environmental compatibility, does not damage the bioactive compounds of the materials, and does not result in the formation of benzopyrene when compared with hexane extraction [14]. Butane is a relatively cheap solvent which has a high solvation power and does not leave toxic residues [15,16]. Extraction is a continuous counter current process which requires lower pressure and temperature [17]. Furthermore, the solvent can be removed completely by system depressurization at a low temperature and can be recovered [11].

Response surface methodology (RSM) represents a collection of mathematical and statistical techniques useful for determining the effects of different variables and optimizing the response variables. The RSM has been successfully utilized for optimization of the seed oil extraction process by other researchers [15,18,19,20].

Researches have recently focused on the pharmacological effects of fenugreek extracts [1,21], lipids [7], proteins [22], saponins [9], gums [23], essential oils [24], and volatile compounds [25] of fenugreek seed. To our knowledge, no reports have been published about the fenugreek seed oil produced by SBE. Thus, the present work is aimed to optimize the SBE process of fenugreek seed oil using RSM, analyze the fatty acids and physicochemical characteristics of the obtained oil, and compare the SBE with ASE on oil yield and fatty acid composition.

## 2. Results and Discussion

### 2.1. Effects of Single Factors

**Figure 1:**
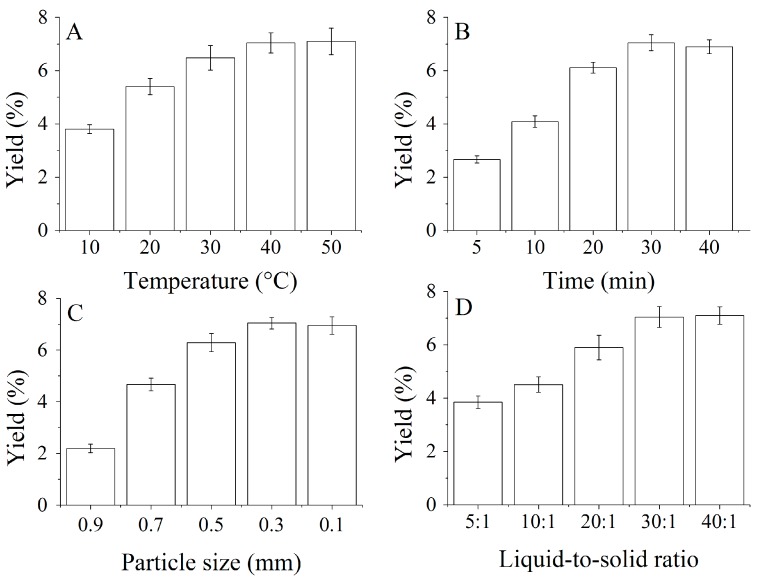
Effects of (**A**) extraction temperature; (**B**) extraction time; (**C**) raw material particle size and (**D**) liquid/solid ratio on the oil yield.

Figure 1 shows the effects of extraction temperature (A), extraction time (B), particle size (C), and liquid/solid ratio (D) on the oil yield of SBE. As inferred from Figure 1A, the oil yield continuously increased from 3.80% to 7.04% as the temperature increased from 10 to 40 °C, however, the oil yield remained constant with further increases in temperature. The density of the butane reduced with the increasing temperature and resulted in the decrease of the oil solubility; however, the pressure of the oil dissolved in subcritical butane increased simultaneously with the increased temperature, which improved the solubility of oil [26]. Therefore, it was concluded that 40 °C was sufficient to achieve the highest oil yield from the fenugreek seed. Similar yield trend as a function of temperature was observed in the extraction of other materials [26]. However, the highest oil yields produced from diverse material were usually achieved at different temperatures due to their distinctive biochemical composition [27].

The oil yield increased as extraction time increased from 5 to 30 min, and reached the maximum at 30 min. The oil yield started to decrease with further increases of the extraction time (Figure 1B). This phenomenon could be explained by a balance being attained between butane and oil over a period of time. Similar trends were also found in other oil extraction processes [15,26]. For yield and processing cost consideration, 30 min were sufficient for the extraction of seed oil. Thus, extraction time of 30 min was selected as the “0” level in the RSM experiments.

As can be seen in Figure 1C, particle size played an important role in the oil yield. The oil yield increased remarkably with the decreased material particle size from 0.9 to 0.3 mm, while below 0.3 mm, the oil yield remained constant. The results can be explained by mass transfer resistances. The extraction of oil from the seeds involved two general kinds of mass transfer resistances; namely, internal mass transfer resistance and external mass transfer resistance. The particle size of the seed powder could obviously affect the oil yield when the internal mass transfer resistance was the dominant resistance of the whole process. However, in the case where external mass transfer resistance is the dominant resistance of the whole process, the particle size of the material will not be able to significantly affect the oil yield [28]. Finally, a particle size of 0.3 mm was selected as the “0” level of particle size in the RSM experiments.

According to the literature, extractor diameter to length ratio (D/L) was another factor that affects the oil yield in the supercritical CO_2_ extraction process [29]. However, it is difficult to achieve in the subcritical extraction process because of the subuliform bottom of the extractor. Therefore, the effect of the liquid/solid ratio on the fenugreek seed oil yield was surveyed in the single-factor experiments’ design. As can be seen in Figure 1D, the fenugreek seed oil yield increased from 3.85% to 7.04% as the liquid/solid ratio increased from 5:1 to 30:1 (mL/g). These results were also suggest that the liquid/solid ratio had a significant positive effect on improving the oil yield when it was under 30:1. The effect was not significant when the liquid/solid ratio was above 30:1. These results would be explained because the contact area between material powder and subcritical butane fluid increased with the increased liquid/solid ratio; hence, more oil was dissolved out from the material. However, no more oil was dissolved out with a continued increase in the liquid/solid ratio. Besides, a higher liquid/solid ratio could cause waste energy and increase costs. Therefore, it must be controlled at a desired level if one expects to achieve a higher oil yield. Thus, the ratio of 30:1 was selected for to further optimize experiments.

### 2.2. Response Surface Optimization of SBE

#### 2.2.1. ANOVA Analysis and the Model Fitting

The ANOVA results for the model of SBE conditions are shown in Table 1. The coefficient of determination (*R*^2^) was used for judging the proportion of variability in the data explained or accounted for by the polynomial model [18]. The model showed a satisfactory *R*^2^ (0.9713), indicating that the model adequately explained the total relationship among the independent variables. The model *F*-value of 26.29 and *p*-value of 0.0001 demonstrated the quadratic polynomial regression model was significant. The *F*-value and *p*-value of the lack of fit were 3.72 and 0.1183, respectively, indicating that the lack of fit of the model was not significant, thereby confirming the goodness-of-fit and suitability of the model.

The adjusted determination coefficient (Adj. *R*^2^) was 0.9343 for the polynomial model, which indicated that the model could work well for further testing of the prediction of the oil yield. The coefficient of variation (CV %) of the model was 2.25% (< 5%), which indicated that the model was reproducible [15].

Table 1 lists the regression coefficients of the intercept, linear, quadratic, and interaction terms of the model. *F*-value and the *p*-value were used in judging the significance of each coefficient. The values of *p* < 0.01, *p* < 0.05, and *p* > 0.05 indicated highly significant, significant, and not significant of the model terms, respectively. This implied that three linear (*X*_1_, *X*_2_, and *X*_3_), two quadratic (*X*_2_^2^ and *X*_3_^2^), and one interaction parameters (*X*_1_*X*_3_) were highly significant model terms. The interaction parameters *X*_1_*X*_2_ and quadratic *X*_1_^2^ were significant (*p* < 0.05) model terms. The interaction parameter *X*_2_*X*_3_ was an insignificant (*p* > 0.05) model term.

A quadratic equation for calculating the oil yield was obtained, and it is provided below as Equation (1):

(1) $$\begin{array}{l} Y\;=\;-\;4.3917+\;0.2362X_{1}+\;0.4373X_{2}-\;5.4022X_{3}-\;0.0022X_{1}X_{2}+\;0.2173X_{1}X_{3}\\ +\;0.0494X_{2}X_{3}-\;0.0026X_{1}{}^{2}-\;0.0054X_{2}{}^{2}-\;10.7163X_{3}{}^{2} \end{array}$$

where *Y* was oil yield, *X*_1_ was extraction temperature, *X*_2_ was extraction time, and *X*_3_ was particle size.

#### 2.2.2. Response Surface Analysis

**Figure 2:**
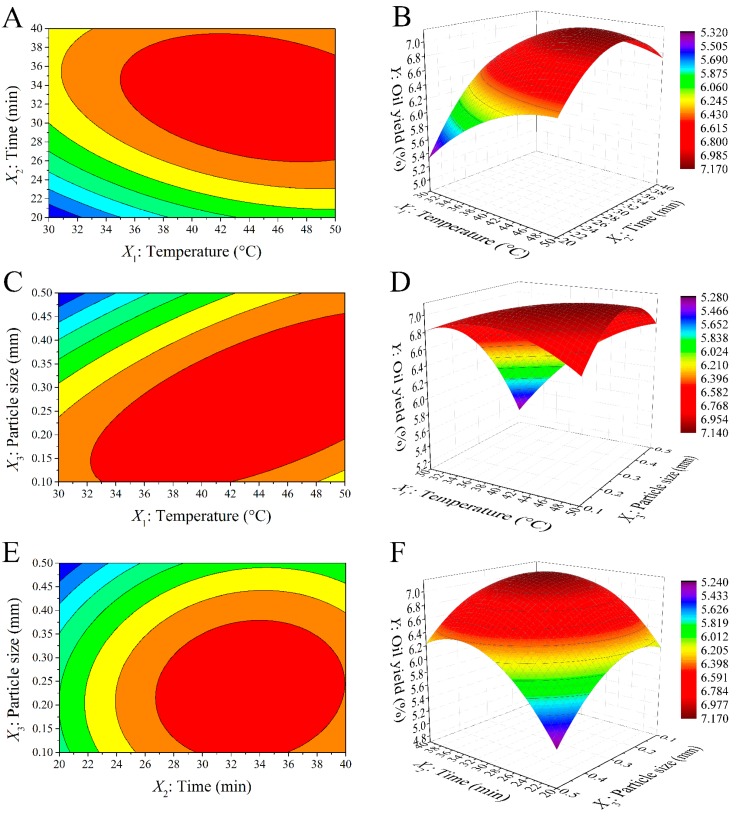
Contour plots (**A**,**C**,**E**) and response surface plots (**B**,**D**,**F**) of the oil yield affected by extraction temperature (°C, *X*_1_), time (min, *X*_2_) and raw material particle size (mm, *X*_3_).

The two-dimensional (2D) contour plots and three-dimensional (3D) response surface plots (Figure 2) were generated by Origin 9.0 software while holding a variable at the “0” level in the quadratic equation. This is the most intuitive way to express the effects of any independent variable on the oil yield [20].

The contour plot and the response surface plot based on extraction temperature and extraction time are shown in Figure 2A,B, while the particle size was kept at 0.3 mm. The oil yield was increased with increasing extraction time until it reached a plateau value at >32.80 min, which indicated that prolonging the extraction time did not result in a further increase in oil yield. The solubility of oil in subcritical butane fluid increased with increasing temperature, which resulted in an increased oil diffusion coefficient. In this study, extraction temperatures <43.24 °C had a positive effect on the oil yield. The results obtained in this study were consistent with the report by Liu et al. [15] which indicated that the temperature had a significant effect on the subcritical fluid extraction of seed oil from *Nitraria tangutorum* oil.

Figure 2C,D show the contour plot and the response surface plot based on changed particle size and extraction temperature at a constant extraction time (30 min). The results imply that the oil yield increased, evidently, with the decrease of particle size from 0.5 to 0.26 mm at low temperatures; however, at the high temperatures, the oil yield had no obvious change as the temperature further increased. The increased oil yield with decreased particle size was attributed to the larger surface area per unit mass, and this resulted in a more effective contact area for the oil to the subcritical butane solvent. Furthermore, the migration speed of the oil dissolving out from the raw material was also increased with the decrease in the particle size. These results were different from previous studies [15,30]. This was mainly attributed to the difference in the type of raw materials.

Figure 2E,F show the effect of extraction time and particle size on the fenugreek seed oil yield at the “0” level of extraction time (30 min). The circular contour plots indicated that the interactions between the two variables were negligible. These results implied that the extraction yield increased with increasing extraction time until reaching a plateau at all particle sizes. The highest oil yield was observed with an extraction time and raw material particle size of approximately 32.80 min and 0.26 mm, respectively. According to literature [26], a maximum yield of red pepper seed oil was obtained at an extraction time of 68.65 min. Similar results have been also obtained for the subcritical fluid extraction of seed oil from *Nitraria tangutorum* [15].

### 2.3. Optimization of Extraction Conditions

The optimal condition of SBE obtained by the optimization option of the Design Expert software was as follows: with butane solvent, a liquid/solid ratio of 30:1, a temperature of 43.24 °C, an extraction time of 32.80 min, and a particle size of 0.26 mm. Under the optimal condition, the predicted value of oil yield was 7.18%. The optimal condition was slightly modified to a particle size of 0.26 mm, an extraction time of 33 min, and a temperature of 43 °C, for convenience purposes. The result revealed that the actual experimental value (7.17%, *n* = 3) was consistent with the predicted value (7.18%). The results confirmed that the response model was reliable and accurate enough to predict the oil yield of SBE.

### 2.4. Comparison of SBE and ASE on Oil Yield and Fatty Acid Composition

For ASE, the procedure conditions were used as described in the methods section. Ultimately, an oil yield of 7.22% was attained (maximum 7.18% for SBE). It can be seen that the two methods had approximately the same oil yield. Moreover, SBE did not use toxic organic solvents and performed under lower pressure and temperature. Therefore, SBE seems to be the preferred method for the extraction of fenugreek seed oil from the aspects of oil yield, energy consumption, and industrial production.

Fatty acid composition and content of the oils obtained by these two methods are shown in Table 2. All the oils extracted were rich in unsaturated fatty acids (UFA) (oleic acid, linoleic acid, and linolenic acid making up from 83.19% to 83.33% of total fatty acids) and low in saturated fatty acids (SFA) (primarily palmitic acid, making up from 9.94% to 10.00% of total fatty acids). A similar result was also found by Sulieman et al. [2].

The oils were rich in linoleic acid (C18:2) (42.71%–42.80%), which was about eight times higher than that of virgin olive oil [31]. Linoleic acid is an important polyunsaturated fatty acid (PUFA), which plays an important role in muscle, brain and nervous system growth. In addition, it benefits the dermal, skeletal, metabolic, and reproductive systems [28]. The oil was also rich in linolenic acid (C18:3) (26.03%–26.15%), which was much higher than that of virgin olive oil (0.60%) [31]. In recent years, linolenic acid (C18:3) was found to have some biological functions, especially strong antitumor activity in vitro [32]. The oleic acid (C18:1) (14.24%–14.40%) content of the oil was lower than that of virgin olive oil (78.20%) [31]. Oleic acid (C18:1) was an important monounsaturated fatty acid (MUFA) which played an important role in the prevention and treatment of cardiovascular disease [33]. The SFA such as, myristic acid (C14:0), palmitic acid (C16:0), stearic acid (C18:0), arachidic acid (C20:0), and lignoceric acid (C22:0) contribute 0.14%, 9.94%–10.00%, 4.68%–4.71%, 1.33%, and 0.59%–0.63%, respectively to the total fatty acids composition. The content of palmitic acid (C16:0) was lower than that obtained by Sulieman et al (11.00%) [2]. The fatty acid profile and high amounts of unsaturated fatty acids, especially polyunsaturated fatty acids, make the fenugreek seed oil a special component for functional applications as edible oil.

It can be concluded that the fatty acid contents of fenugreek seed oils obtained by SBE and ASE were very similar, while a small amount of erucic acid (C22:1) (0.20%) was found in the unsaturated fatty acids of the oil extracted by ASE. This phenomenon can be attributed to the extraction kinetics, and the extraction efficiency of fatty acids were positively influenced by extraction solvent, high-pressure, and high-temperature used by ASE [12].

### 2.5. Physicochemical Characteristics of the Oil

The physicochemical characteristics of fenugreek seed oil extracted by SBE at the optimal conditions are shown in Table 3. At room temperature, the oil was yellow (yellow units 70.647, red units 9.516, blue units 0.586) and had a refractive index of 1.479, which is equal to soybean oil (1.477) and corn oil (1.473) [34]. The high refractive index value of the oil was because of a high content of UFA, which proved that the fenugreek seed oil had the qualities of edible oil. The relative density of the oil was 0.922. The acid value refers to the combined effect of all the fatty acids that make up the glyceride molecule, which represents the total acidity of the oil. The acid value of the oil was 6.413 mg/g oil, which would reach the allowable limits for edible oils through an appropriate refining process [19]. The high iodine value (148.564 g/100 g of oil) was because of its low content of SFA. The high saponification value (190.277 mg KOH/g of oil) of the oil indicated a high content of low molecular weight triacylglycerols in the oil. These results were in agreement with previous research [8]. The seed oil contained 3.790% unsaponifiable matter, which was higher than the 1% unsaponifiable matter standard suggested by Fontanel [35]. The unsaponifiable matter of the seed oil includes tocopherols, sterols, triterpenic alcohols, hydrocarbons, and aliphatic alcohols [36]. The peroxide value of the oil was 0.627 meq. O_2_/kg oil, the value was relatively lower because the oxidization could be avoided during the course of SBE [26]. In relation to tocopherol, only α-tocopherol (16.460 mg·100 g^−1^) was identified from the oil (Table 3). This value was higher than that of virgin olive oil (14.600 mg·100 g^−1^) [31]. Therefore, these physicochemical characteristics demonstrated that the oil extracted in the present study was an excellent candidate for use as a functional edible oil.

Oxidation of lipids was a major reason for the metamorphism of edible oils; during this process, the main oxidation products of PUFAs in seed oil decompose promptly then form many different kinds of volatile and non-volatile secondary oxidation products. The oxidation process observably changes the chemical, sensory, and nutritional properties of the oil. The induction time (IT) of fenugreek seed oil was 2.850 h at 120 °C (Table 3). The IT of the oil was shorter than that of virgin olive oil (20.900 h at 120 °C). This was probably because the oil contains a higher amount of PUFAs (linoleic acid 42.80%, linolenic acid 26.15%) than olive oil (linoleic acid 4.80%, linolenic acid 0.60%) [31].

**Figure 3:**
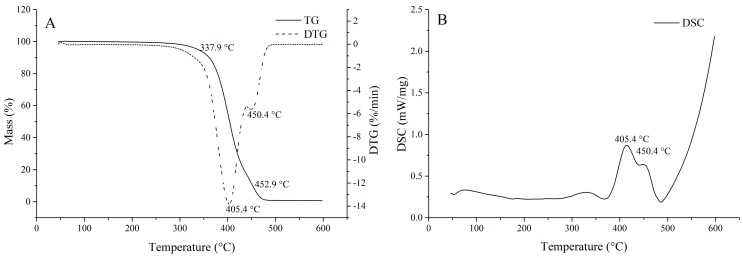
(**A**) Thermal gravity (TG)/differential thermogravimetry (DTG) and (**B**) differential scanning calorimetry (DSC) curves of fenugreek seed oil obtained by SBE.

The thermal gravity analysis (TGA) and differential thermogravimetry (DTG) data of the oil are given in Figure 3A. A 5% mass loss occurred at 337.9 °C, and a 90% mass loss appeared at 452.9 °C for the oil. The result suggests that the oil obtained by SBE is thermally stable and can be used as high-temperature frying oil. The thermal gravity (TG)/DTG curves of the oil showed two mass-loss stages, at 405.4 °C and 450.4 °C, respectively. These stages were connected with volatilize and/or combustion of the triglycerides. In the thermal decomposition stages, it was found that the first stage was because of the decomposition of PUFAs, this was the most important step to determine the thermal stability order of edible oils [37]. The second thermal decomposition stage was attributed to MUFAs decomposition. Double bonds were broken and resulted in saturation of triglyceride molecules during this process.

Differential scanning calorimetry (DSC) curves of the oil presented similar profiles, with endothermic and exothermic transitions, as shown in Figure 3B. Exothermic transitions were a response to the polymerization of fatty acids, whereas, endothermic transitions were potentially attributed to thermal decomposition of the fatty acids [38]. These results were in agreement with the TG/DTG curves (Figure 3A). The enthalpies calculated from DSC curves were related to polymerization and decomposition molar enthalpies. The results indicated that these enthalpies were correct in connection with the fatty acid composition of the oil.

## 3. Materials and Methods

### 3.1. Materials

Fenugreek seed was purchased from a regional pharmacy, located at Zhengzhou, China. Samples were ground into powder with a Wiley Mill (Thomas Scientific, Philadelphia, PA, USA) and passed through different sieve sizes according to experimental design. The seed powder was air-dried for 24 h at 80 °C and then stored at 4 °C for further use. Butane was purchased from Puyang Longyu Chemical Co., Ltd. (Henan, China). Other chemicals and solvents used for this study were of either analytical or chromatographic grade, which were all purchased from Fisher Scientific Chemical (Loughborough, UK) or Sigma Aldrich (Steinheim, Germany).

### 3.2. Oil Extraction

**Figure 4:**
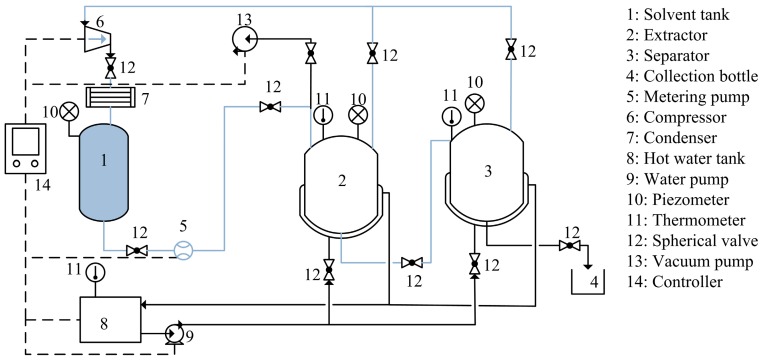
Schematic diagram of the SBE process.

SBE was performed using the apparatus (Henan Subcritical Biological Technology Co., Ltd., Anyang, China) (Figure 4). For SBE, 50 g of sample powder was loaded into the extractor. The filled extractor was then vacuum sealed to remove atmospheric oxygen, which could cause oxidation of the oil during extraction. The butane was introduced into the extractor through a metering pump in the form of subcritical fluid. The temperature was automatically controlled by a controller unit according its setting. At the end of the extraction, the liposoluble extract reached the separator, and after the solvent was removed by evaporation, the extracted oil was gathered. The mass of the oil was measured with an analytical balance and then stored at 4 °C in a refrigerator for further analysis.

ASE was performed in an ASE200 Accelerated Solvent Extractor (Dionex, Sunnyvale, CA, USA). Samples (5 g of fenugreek seed powder was mixed with 15 g diatomite) were placed in a 33 mL extraction vessel. The extractions were programmed for 3 cycles with petroleum ether solvent, 5 min of heating, 30 min of extraction at a temperature of 100 °C and a pressure of 1500 Psi (60% rinse volume, 5 s rinsing). The solvent was evaporated under a stream of N_2_. The oil was weighed and then stored at 4 °C refrigerator for further analysis.

The oil yield was calculated as follows, namely Equation (2):

(2) $$\mathrm{The}\;\mathrm{oil}\;\mathrm{yield}\;(\mathrm{wt}\;\%)=f_{0}-\;f_{1}$$

where *f*_0_ and *f*_1_ are the fat contents of raw materials (wt %) before and after extraction. The American Oil Chemists’ Society (AOCS) official method (2009) Ba 3-38 was used to determine the fat content values of raw materials.

### 3.3. Experimental Design

#### 3.3.1. Single-Factor Experiments

Previous studies suggested that temperature always showed the most significant effect on the yield and properties of oil among all the variables examined [26]. Therefore, the effect of extraction temperature was first studied, rather than other variables that could influence the oil yield, with an aim to select the most suitable temperature for the SBE of oil from fenugreek seed. In all experiments, extraction time was maintained at 30 min, particle size was 0.3 mm, and the liquid/solid ratio was 30:1 with varying temperatures from 10 to 50 °C. Extraction time was another important factor that affects the extraction process. The influences of extraction time on fenugreek seed oil yield were evaluated when other factors were set as follows: particle size 0.3 mm, temperature 40 °C; and liquid/solid ratio 30:1. To investigate the effects of the liquid/solid ratio on the fenugreek seed oil yield, the extraction process was carried out using different liquid/solid ratios (5:1, 10:1, 20:1, 30:1, and 40:1). Other extracting variables were fitted as follows: particle size 0.3 mm, extraction time 30 min, and extraction temperature 40 °C. The effect of particle size on extraction efficiency was evaluated at a given liquid/solid ratio (30:1), extraction time (30 min), and extraction temperature (40 °C).

#### 3.3.2. Response Surface Methodology

Based on the preliminary single-factor experiments, RSM with Box-Behnken design (BBD) was applied to statistically optimize the process variables for subcritical butane extraction of fenugreek seed oil. The BBD was specifically selected because it requires fewer runs than a central composite design (CCD) in cases of three variables. 

Three independent variables, i.e., extraction temperature (°C, *X*_1_), extraction time (min, *X*_2_), and particle size (mm, *X*_3_), were selected to optimize the extraction process. Oil yield (*Y*) was taken as the response variable in the optimization experiments. The BBD matrix and the experimental and predicted oil yields are shown in Table 4.

The whole design matrix consisted of 17 experimental points with four replicates at the center points to evaluate the pure error. The behavior of the matrix was explained by the following second-order polynomial regression equation:

(3) $$Y=\beta_{0}+\sum_{i=1}^{3}\beta_{i}X_{i}+\sum_{i=1}^{3}\beta_{ii}X_{i}^{2}+\sum_{i=1}^{2}\sum_{j=i+1}^{3}\beta_{ij}X_{i}X_{j}$$

where *Y* presents the response variables, and *β*_0_, *β_i_*, *β_ii_*, and *β_ij_* were the regression coefficients of variables for constant, linear, quadratic, and interaction terms, respectively. *X_i_* and *X_j_* were the coded levels of independent variables (*i* ≠ *j*). *X_i_*^2^ and *X_i_X_j_* were the quadratic and interaction terms, respectively.

### 3.4. Characterization of Seed Oil

A Lovibond tintometer (PFX880, Tintometer Ltd., Salisbury, UK) was used to determine the color of the fenugreek seed oil. The standards of ISO (International Organization for Standardization) were used in the determination of refractive index (ISO 6320, 2000), oil density (ISO 3675, 1998), acid value (ISO 660, 2009), peroxide value (ISO 3960, 2007), iodine value (ISO 3961, 2013), saponification value (ISO 3657, 2013), and unsaponifiable matter (USM) (ISO 3596, 2000). The tocopherol content of fenugreek seed oil was determined according to the method described by Santos et al. [14].

The oxidative stability was determined by Rancimat (Metrohm 743, Herisan, Switzerland) according to ISO 6886: 2006. The oil samples (3 g) were placed in the Rancimat apparatus at a temperature of 120 °C, under a constant air flow (20 L·h^−1^). The induction time (IT) was recorded automatically by the apparatus software.

Thermal properties were analyzed with a Netzsch STA 449C thermal analyzer (Netzsch Group, Selb, Germany) using sample mass of about 10 mg, in nitrogen atmosphere (50 mL·min^−1^), using aluminum crucibles, in a temperature range of 45–600 °C with a heating rate of 10 °C·min^−1^. With the help of the simultaneous thermal analyzer, the data of the TG/DTG and DSC were obtained simultaneously.

### 3.5. Determination of Fatty Acid

Fatty acid methyl esters (FAME) were prepared by the methylation of lipids according to the procedure described in detail in our previous work [26]. Prepared fatty acid methyl esters were analyzed in accordance with the method used by Da Porto et al [10]. The analysis was carried out on a gas chromatography system (7890A, Agilent Co., Santa Clara, CA, USA) coupled to a flame ionization detector (FID) and a capillary column (HP-88, 100 m × 0.25 mm × 0.20 μm). For qualitative analysis, retention times of fatty acid curves were compared with those of standard methyl esters (Sigma Aldrich Co., Steinheim, Germany). For quantitative analysis, fatty acid content was determined by measuring the peak area.

### 3.6. Statistical Analyses

All experiments were performed in triplicate, and all the results were expressed as mean value ± SD (standard deviation). The results were analyzed by one-way analysis of variance (ANOVA). Data collected from the SBE were processed by the software Design-Expert 8.0.6 (Stat-Ease, Inc., Minneapolis, MN, USA) and Origin 9.0 (OriginLab, Northampton, MA, USA).

## 4. Conclusions

The optimization of the SBE of oil from fenugreek seed was performed with a statistical method based on the RSM in order to identify and quantify the variables (i.e., extraction temperature, extraction time, and particle size) which would maximize the fenugreek seed oil yield. The results demonstrated that the optimal conditions for the extraction of fenugreek seed oil by SBE were as follows: liquid/solid ratio of 30:1, extracting temperature of 43.24 °C, extracting time of 32.80 min, and particle size of 0.26 mm. Fatty acid composition of the oils obtained by SBE and ASE showed negligible differences, and both the oils were rich in UFAs (mainly linoleic acid, linolenic acid, and oleic acid). The oil also showed its desirable thermal and oxidative stability. From the results, it was found that SBE is effective for the preparation of fenugreek seed oil, and the oil could be used as edible oil.

## Figures and Tables

**Table 1:** Analysis of variance for the quadratic polynomial mode.

Source ^1^	Coefficient Estimate	Standard Error	Sum of Squares	DF	Mean Square	*F*-Value	*p*-Value ^2^
Model	N/A	N/A	6.41	9	0.71	26.29	0.0001
Intercept	7.04	0.074	N/A	N/A	N/A	N/A	N/A
*X*_1_	0.31	0.058	0.77	1	0.77	28.34	0.0011
*X*_2_	0.39	0.058	1.24	1	1.24	45.95	0.0003
*X*_3_	−0.33	0.058	0.88	1	0.88	32.46	0.0007
*X*_1_*X*_2_	−0.22	0.082	0.19	1	0.19	6.95	0.0336
*X*_1_*X*_3_	0.43	0.082	0.76	1	0.76	27.90	0.0011
*X*_2_*X*_3_	0.099	0.082	0.039	1	0.039	1.44	0.2692
*X*_1_^2^	−0.26	0.080	0.28	1	0.28	10.25	0.0150
*X*_2_^2^	−0.54	0.080	1.24	1	1.24	45.88	0.0003
*X*_3_^2^	−0.43	0.080	0.77	1	0.77	28.56	0.0011
Residual	N/A	N/A	0.19	7	0.027	N/A	N/A
Lack of Fit	N/A	N/A	0.14	3	0.047	3.72	0.1183
Pure Error	N/A	N/A	0.050	4	0.012	N/A	N/A
SD	0.16	N/A	*R*^2^	0.9713	N/A	N/A	N/A
Mean	6.47	N/A	Adj. *R*^2^	0.9343	N/A	N/A	N/A
CV (%)	2.55	N/A	Pred. *R*^2^	0.6497	N/A	N/A	N/A
PRESS	2.31	N/A	Adeq. Precision	14.233	N/A	N/A	N/A

DF, degrees of freedom; SD, standard deviation; CV, coefficient of variation; N/A, not applicable. ^1^ *X*_1_, extraction temperature; *X*_2_, extraction time; *X*_3_, particle size. ^2^ *p* < 0.01 indicates high statistical significance; *p* < 0.05 indicates statistical significance; *p* > 0.05 indicates statistical non-significance.

**Table 2:** Fatty acid composition of the fenugreek seed oils extracted by different methods.

Fatty Acid	SBE ^1^ (%)	ASE ^2^ (%)
Myristic acid (C14:0)	0.14 ± 0.01	0.14 ± 0.01
Palmitic acid (C16:0)	10.00 ± 0.16	9.94 ± 0.13
Stearic acid (C18:0)	4.71 ± 0.13	4.68 ± 0.20
Oleic acid (C18:1)	14.24 ± 0.08	14.40 ± 0.14
Linoleic acid (C18:2)	42.80 ± 0.11	42.71 ± 0.13
Arachidic acid (C20:0)	1.33 ± 0.03	1.33 ± 0.04
Linolenic acid (C18:3)	26.15 ± 0.20	26.03 ± 0.12
Behenic acid (C22:0)	0.63 ± 0.01	0.59 ± 0.03
Erucyl alcohol (C22:1)	ND ^3^	0.20 ± 0.02

^1^ SBE, subcritical butane extraction; ^2^ ASE, accelerated solvent extraction; ^3^ ND, not detected.

**Table 3:** Physicochemical characteristics of fenugreek seed oil.

Properties		Values
Color	red units	9.516 ± 0.133
	yellow units	70.647 ± 1.612
	blue units	0.586 ± 0.049
Refractive index		1.479 ± 0.233
Relative density		0.922 ± 0.021
Acid value (mg/g oil)		6.413 ± 0.196
Iodine value (g/100 g oil)		148.564 ± 2.025
Saponification value (mg KOH/g oil)		190.277 ± 2.391
Unsaponifiable matter (%)		3.790 ± 0.215
Peroxide value (meq. O_2_/kg oil)		0.627 ± 0.033
Induction time (h, 120 °C)		2.850 ± 0.081
α-Tocopherol (mg·100 g^−1^)		16.460 ± 0.667

**Table 4:** Box-Behnken design (BBD) matrix and the response values for the oil yield of fenugreek seed.

RUN	Independent Variable			Oil Yield (%)	
	*X*_1_ (Temperature, °C)	*X*_2_ (Time, min)	*X*_3_ (Particle Size, mm)	Experimental	Predicted
1	50	30	0.1	6.75 ± 0.26	6.57
2	40	30	0.3	6.94 ± 0.31	7.04
3	40	20	0.5	5.33 ± 0.23	5.25
4	50	30	0.5	6.74 ± 0.12	6.77
5	40	30	0.3	6.93 ± 0.28	7.04
6	40	30	0.3	7.06 ± 0.36	7.04
7	40	30	0.3	7.12 ± 0.11	7.04
8	30	20	0.3	5.43 ± 0.09	5.32
9	50	20	0.3	6.33 ± 0.27	6.38
10	50	40	0.3	6.63 ± 0.30	6.73
11	40	30	0.3	7.18 ± 0.28	7.04
12	40	40	0.1	6.62 ± 0.17	6.70
13	30	30	0.5	5.10 ± 0.10	5.28
14	30	30	0.1	6.85 ± 0.25	6.82
15	30	40	0.3	6.59 ± 0.14	6.55
16	40	20	0.1	5.98 ± 0.33	6.11
17	40	40	0.5	6.37 ± 0.21	6.23
